# Genomic Characterizations of Clade III Lineage of *Candida auris*, California, USA 

**DOI:** 10.3201/eid2704.204361

**Published:** 2021-04

**Authors:** Travis K. Price, Ruel Mirasol, Kevin W. Ward, Ayrton J. Dayo, Evann E. Hilt, Sukantha Chandrasekaran, Omai B. Garner, Annabelle de St Maurice, Shangxin Yang

**Affiliations:** University of California, Los Angeles, California, USA

**Keywords:** yeast, fungal infections, *Candida auris*, Los Angeles, California, clade III, outbreak, genomic characterization, United States, fungi, antimicrobial resistance, *Candida*

## Abstract

*Candida auris* is an emerging multidrug-resistant yeast. We describe an ongoing *C. auris* outbreak that began in October 2019 in Los Angeles, California, USA. We used genomic analysis to determine that isolates from 5 of 6 patients belonged to clade III; 4 isolates were closely related.

*Candida auris* was isolated from a patient in Tokyo, Japan in 2009 (*1*), although clinical isolates have been retrospectively identified from as early as 1996 (*2*). Since then, bloodstream and other invasive infections caused by *C. auris* have been reported worldwide (*3*–*5*). Many strains of *C. auris* are multidrug-resistant; some strains require elevated MICs to azoles, echinocandins, and polyenes. In 2019, the US Centers for Disease Control and Prevention (CDC) listed *C. auris* as an urgent threat to public health (*6*), highlighting the need for active surveillance and appropriate infection prevention.

Whole-genome sequencing (WGS) and phylogenetic analyses have revealed >4 major clades of *C. auris*; each clade covers a distinct geographic area, giving *C. auris* a global distribution (*7*,*8*). Researchers have documented several *C. auris* outbreaks in the United States, mostly caused by strains belonging to clades I and IV (*9*). We describe several cases of *C. auris* colonization and infection in patients of long-term acute-care (LTAC) facilities in and around Los Angeles, California, USA. 

## The Study

We screened patients at high risk for drug-resistant infections who were transferred to University of California, Los Angeles (UCLA)–affiliated hospitals from LTAC and skilled nursing facilities (SNFs). We analyzed swab samples of patients’ axilla and groin and yeast isolates from positive fungal culture of clinical specimens using PCR selective for the ITS2 region of the *C. auris* genome. We conducted antifungal susceptibility testing using broth microdilution; WGS using Illumina MiSeq (Illumina, https://www.illumina.com); and k-mer and single-nucleotide polymorphism (SNP) analyses using CLC Genomics Workbench (QIAGEN, https://www.qiagen.com) and Geneious Prime (Geneious, https://www.geneious.com) (Appendix 1). 

During September 2019–September 2020, we screened 113 patients using in-house PCR selective for *C. auris* according to Los Angeles County Public Health and CDC guidelines (Appendix 1). Six patients tested positive for *C. auris* with cycle threshold (C_t_) values of 22.6–39.7 (Table 1). Patient A tested positive in October 2019; patients B–F tested positive during July–September 2020.

**Table 1:** Characteristics of patients with *Candida auris* infection, Los Angeles, California, USA, 2019–2020*

Patient	Date of positive PCR	Cycle threshold	*C. auris* isolate (specimen type)	Approximate age, y	Clinical history	Current signs, symptoms, and diagnosis
A	2019 Oct 8	22.6	UCLA_A1 (inguinal–axillary); UCLA_A2 (tracheal, deemed colonization)	65	Coronary artery disease, stroke, chronic respiratory fracture, tracheostomy and ventilator dependence, gastrostomy tube dependence, urinary incontinence, multiple ulcers, heart failure, atrial fibrillation, and previous carbapenem-resistant *Enterobacteriaceae* bacteremia. This patient had a prior history of *C. auris* colonization at the long-term acute-care facility.	Septic shock caused by methicillin-resistant *Staphylococcus aureus* bacteremia and multifocal pneumonia. *C. auris*, *Candida albicans*, and *Candida parapsilosis* were isolated from tracheal suction culture.
B	2020 July 28	39.5	Not isolated†	45	Anoxic brain injury caused by MRSA endocarditis and pulseless electrical activity arrest, stroke, and gastrostomy tube dependence.	Hemoptysis, upper gastrointestinal bleeding, hypotension, and tachycardia. MRSA, *Escherichia coli*, *Providencia stuartii*, *Proteus mirabilis*, and *Acinetobacter baumanii* grew on blood cultures. *Candida glabrata* grew on lower respiratory culture.
C	2020 Aug 12	22.6	UCLA_C1 (inguinal–axillary)	65	Hypertension, hyperlipidemia, intracranial hemorrhage and ventriculoperitoneal shunt, tracheostomy, and gastrostomy tube dependence.	Respiratory failure caused by pulmonary edema. *P. mirabilis* grew on urine cultures.
D	2020 Aug 19	39.7	UCLA_D1 (inguinal–axillary)	55	Hypertension, hyperlipidemia, type 2 diabetes, aplastic anemia, stroke, pulmonary embolism, pneumothorax, and coronavirus disease–related pneumonia causing respiratory failure, tracheostomy, and gastrostomy tube dependence.	Elevated liver enzymes and gastrointestinal bleeding complicated by *Enterococcus* bacteremia and *E. coli* urinary tract infection.
E	2020 Aug 31	28.3	UCLA_E1 (inguinal–axillary)	65	Hypertension, hyperlipidemia, tracheostomy, and gastrostomy tube dependence.	Worsening generalized weakness possibly caused by chronic intermittent demyelinating polyneuropathy.
F	2020 Sep 3	30.6	UCLA_F1 (pleural fluid, active infection)	85	Subarachnoid hemorrhage, tracheostomy, gastrostomy tube dependence, stage IV sacral decubitus ulcer, and chronic kidney disease. This patient had a prior history of *C. auris* colonization at the long-term acute-care facility.	Bronchopulmonary fistula. *C. auris*, *Pseudomonas aeruginosa*, and *Enterococcus faecalis* grew on pleural fluid cultures.

*MRSA, methicillin-resistant *Staphylococcus aureus*. †*C. auris* was not isolated from the inguinal–axillary surveillance swab of patient B.

The 6 patients were residents of 4 LTAC facilities in Los Angeles County. All 6 had a history of tracheostomy. Patients A and F had prior history of *C. auris* colonization; patient F had active infection of a bronchopulmonary fistula. Patient D had *C. auris* and severe acute respiratory syndrome coronavirus 2 co-infection (Table 1). We cultured *C. auris* isolates from inguinal and axillary swab samples of patients A, C, D, and E; pleural fluid of patient F; and tracheal aspirate of patient A. The sample from patient A produced few colonies; we treated the patient for bacterial pneumonia. We were not able to isolate *C. auris* from patient B (C_t_ = 39.5).

All *C. auris* isolates were resistant to amphotericin B (MIC = 2 μg/mL) and fluconazole (MIC >64 μg/mL) but susceptible to echinocandins (Table 2). We conducted k-mer analysis using 261 *C. auris* sequences available on GenBank, most of which were described previously (*10*) (Appendix 2 Table 1). All 6 UCLA isolates belonged to clade III (Appendix 2 Table 1). We conducted a phylogenetic analysis of clade III isolates using k-mers (Appendix 1 Figure).

**Table 2:** Antifungal susceptibility results for *Candida auris* isolates, Los Angeles, California, USA, 2019–2020*

Antifungal	MIC, μg/mL (interpretation)†					
	UCLA_A1	UCLA_A2	UCLA_C1	UCLA_D1	UCLA_E1	UCLA_F1
Amphotericin B	2 (R)	2 (R)	2 (R)	2 (R)	2 (R)	2 (R)
Fluconazole	>64 (R)	>64 (R)	>64 (R)	>64 (R)	>64 (R)	>64 (R)
Voriconazole	2	1	1	0.5	0.5	2
Itraconazole	1	1	0.5	0.25	0.25	1
Posaconazole	0.06	0.12	≤0.03	≤0.03	0.06	0.06
Anidulafungin	0.5 (S)	0.12 (S)	0.25 (S)	0.12 (S)	0.06 (S)	1 (S)
Caspofungin	0.5 (S)	0.12 (S)	0.5 (S)	0.5 (S)	0.12 (S)	0.5 (S)
Micafungin	0.25 (S)	0.25 (S)	0.25 (S)	0.12 (S)	0.25 (S)	0.25 (S)

*I, intermediate; R, resistant; S, susceptible. †MIC testing was conducted on panels prepared in-house in accordance with Clinical and Laboratory Standards Institute guidelines (https://standards.globalspec.com/std/10266416/CLSI%20M27). Interpretive breakpoints were defined by the CDC Antifungal Susceptibility Testing and Interpretation guidelines for *C. auris* (https://www.cdc.gov/fungal/candida-auris/c-auris-antifungal.html), which were adapted from interpretive criteria for closely related *Candida* spp*.* Tentative breakpoints for the following antifungal drugs were: amphotericin B (>2 μg/mL), fluconazole (>32 μg/mL), anidulafungin (>4 μg/mL), caspofungin (>2 μg/mL), and micafungin (>4 μg/mL).

In the United States, researchers have identified isolates belonging to all 4 clades; although these isolates show geographic relationships (*9*), clade I is predominant across the country. Clade III isolates have been identified in Indiana, Texas (*11*), and Florida. We conducted a k-mer–based phylogenetic analysis of *C. auris* isolates in the United States (Figure). SNP analysis showed that 5 of the UCLA isolates were closely related (3–12 SNPs); isolate F1 was genetically distinct (77–79 SNPs). All 6 isolates were distinct from isolates from Indiana (65–139 SNPs) and Florida (47–117 SNPs) (Appendix 1 Table 1).

**Figure:**
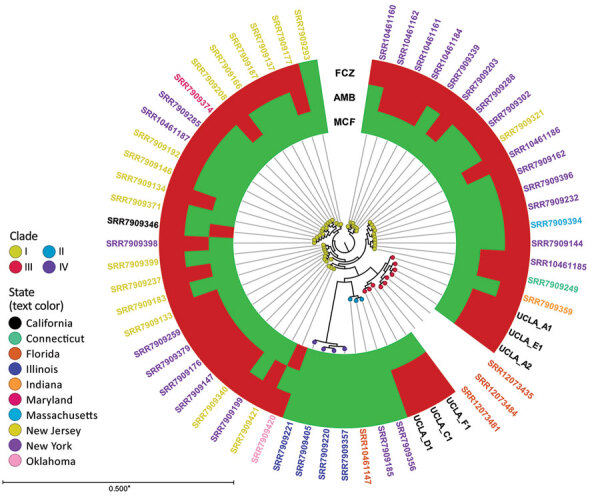
K-mer analysis of *Candida auris* isolates, United States, 2009–2020. K-mer analysis was conducted with CLC Genomics Workbench (QIAGEN, https://www.qiagen.com) using genome sequences from patients in Los Angeles, California, USA during 2019–2020 (i.e., UCLA_A1, UCLA_A2, UCLA_C1, UCLA_D1, UCLA_E1, and UCLA_F1) and 55 publicly available *C. auris* strains in GenBank (Appendix 2 Table 2). Each node represents a unique isolate. Node color indicates clade. The color of the isolate name (i.e., label text color) indicates state of origin. The metadata shows the susceptibility of each isolate (if available) to fluconazole (FCZ), amphotericin B (AMB), and micafungin (MCF); red indicates resistant, green indicates susceptible. Asterisk indicates that branches shorter than 0.0050 are shown as 0.0050.

We also analyzed the sequences of 2 genes associated with antifungal resistance: *erg11* (lanosterol 14-α demethylase) and *fks1* (subunit of 1,3-β-D-glucan synthase). Sequences of *erg11* were identical among all isolates, with 99.6% pairwise nucleotide identity to the reference (GenBank accession no. CP043531) and 2 amino acid substitutions: V125A and F126L (Appendix 1 Table 2). Mutations at aa 126 are associated with increased azole resistance in *C. auris* (*7*) and are a common feature of clade III isolates (*12*). The F126L mutation appears to be exclusive to clade III (*10*). These findings are consistent with results of antifungal susceptibility testing, which showed that all isolates were resistant to fluconazole (Table 2). Sequences of *fks1* were identical in 5 isolates (A1, A2, C1, D1, E1), with 99.9% pairwise nucleotide identity to the reference (GenBank accession no. CP043531); these isolates had 1 amino acid substitution: I1572L (Appendix 1 Table 2). Isolate F1 had the same substitution in addition to I1095L. All isolates had a wild-type serine at aa 639; mutations at this location are linked to echinocandin resistance in *C. auris* (*13*). All isolates were susceptible to caspofungin, micafungin, and anidulafungin.

## Conclusions

To identify and prevent the spread of *C. auris* in this hospital system, we used an in-house PCR to screen patients for this pathogen. WGS of isolates from patients transferred from LTAC facilities revealed that these isolates are closely related, suggesting an ongoing outbreak with community spread in the Los Angeles area.

The isolates described here were all resistant to fluconazole and amphotericin B but susceptible to echinocandins according to the CDC tentative breakpoints (https://www.cdc.gov/fungal/candida-auris/c-auris-antifungal.html). In addition, all isolates had an F126L mutation in the *erg11* gene, which is unique to clade III strains and associated with fluconazole resistance (*10*).

Patient D was admitted to an SNF after complications from pneumonia caused by coronavirus disease (COVID-19). Few cases of *C. auris* and COVID-19 co-infection have been reported (*14*,*15*). After COVID-19 infection, patient D had multiple complications requiring a tracheostomy and enteral feeding tube; the patient was subsequently transferred to an LTAC for rehabilitation. A substantial portion of adult patients who recover from severe COVID-19 have long-term sequelae and might require admission to SNFs or LTACs. Therefore, the COVID-19 pandemic might lead to increased transmission of *C. auris* in SNFs because of increased admissions and shortages of personal protective equipment. During critical shortages, CDC guidelines permit extended use of isolation gowns for patients who are known to be infected with the same infectious disease if there are no additional known coinfections transmitted through contact (https://www.cdc.gov/coronavirus/2019-ncov/hcp/non-us-settings/emergency-considerations-ppe.html#ppe-specific-strategies). To encourage appropriate use of personal protective equipment and prevent transmission, it is essential that facilities screen patients for *C. auris*. 

One limitation of this study is the lack of additional epidemiologic history of the patients, especially in the context of travel-related exposures. The ability to track cases to a location with known outbreaks of clade III *C. auris* strains is essential to determining the origin of the current outbreak. Further investigation is needed to explain why patient F had a genetically distinct isolate, suggesting a separate introduction. 

In conclusion, we identified a unique clade III *C. auris* strain in an ongoing outbreak in LTAC facilities since 2019. These findings indicate active community spread of multidrug-resistant *C. auris* in the Los Angeles area.

Appendix 1 Additional information on methods for genomic characterizations of clade III lineage of *Candida auris*, California, USA.

Appendix 2 Sequences analyzed in k-mer analysis of *Candida auris* isolates, California, USA.
