# Supplementary material for: Genomic Characterizations of Clade III Lineage of Candida auris, California, USA
Source: Emerg Infect Dis. 2021 Apr;27(4):1223–7. doi: 10.3201/eid2704.204361 (PMC8007294; doi:10.3201/eid2704.204361)
Supplement: Appendix 1 — Additional information on methods for genomic characterizations of clade III lineage of Candida auris, California, USA. [file 20-4361-Techapp-s1.pdf]

Article DOI: https://doi.org/10.3201/eid2705.204361

Genomic Characterizations of Clade III Lineage of *Candida auris*, California, USA

Appendix 1

Appendix Methods

Patient Screening Criteria

Patients were screened for *Candida auris* based on Los Angeles County Department of Public Health and U.S. Centers for Disease Control and Prevention guidance for patients at high risk of colonization. Screening recommendations were based on prevalence of *C. auris*; however, at the time of publication patients were screened if: they were a resident of a long-term acute-care or skilled nursing facility that had an ongoing *C. auris* outbreak; if they had an overnight stay in a hospital outside of the United States or in New York, New Jersey, or Illinois (i.e., states where *C. auris* outbreaks have been reported) in the past 12 months; if they had a history of carbapenem-resistant *Enterobacteraciae* infection; or if they had a known history of *C. auris* or had been in contact with a person with *C. auris*. Patients with a positive *C. auris* test were reported back to the facility and to public health.

Candida auris PCR

Inguinal/axillary surveillance swab samples or pure isolates of suspected *C. auris* growth were extracted using the EZ1 Tissue Extraction Kit (QIAGEN, https://www.qiagen.com). Specimens were pelleted and resuspended in 500µL G2 lysis buffer in a tube with 0.5 mm glass beads (Bertin Technologies, https://bertin-technologies.com); specimens were then heated at 95°C for 10 min and placed in a Precellys 24 homogenizer (Bertin Techologies) using the following settings: 6000 rotations per min, 60 s for 3 cycles with 5 s rest. After bead beating, specimens were centrifuged at 5000 rotations per min for 1 minute and 200µL of supernatant was transferred to the EZ1 DNA Tissue Kit Sample Tube. 5µL of the Simplexa Extraction and Amplification Control DNA (DiaSorin Molecular LLC, https://molecular.diasorin.com) was then

added to each sample and extracted using the EZ1 Advanced XL (QIAGEN) using the following settings: 200µL loading volume and 100µL elution volume.

The PCR reaction was conducted using the DiaSorin Molecular LIAISON MDX (previously known as the 3M Integrated Cycler). 5µL of eluted DNA was added to 5µL reaction mix consisting of the following: 2.5X TA Master Mix, SEAC DNA Primer Pair, *C. auris* Primer Pair (DiaSorin Molecular LLC). The PCR was selective for the ITS2 region of *C. auris*. The cyclin parameters were kept consistent with the manufacturer's instructions. A cycle threshold cutoff of 40 was used. An internal laboratory validation showed 100% sensitivity and specificity with a limit of detection of 10^2 CFU/mL (manuscript under review).

Antifungal Susceptibility Testing

Antifungal susceptibility testing by broth microdilution was conducted on panels prepared in-house according to Clinical and Laboratory Standards Institute standards (https://standards.globalspec.com/std/10266416/CLSI%20M27). The U.S. Centers for Disease Control and Prevention tentative MIC breakpoints were used for interpretation of the following drugs: amphotericin B (≥2µg/mL), fluconazole (≥32µg/mL), anidulafungin (≥4µg/mL), caspofungin (≥2µg/mL), and micafungin (≥4µg/mL). Voriconazole, itraconazole, and posaconazole were also tested but no interpretative criteria for *C. auris* are available.

Whole Genome Sequencing

Specimens from patients with positive *C. auris* surveillance PCR results were sub-cultured on inhibitory mold agar and CHROMagar Candida (Becton, Dickinson and Company, https://www.bd.com); yeast isolates were identified using the VITEK MS matrix-assisted laser desorption/ionization time-of-flight system (bioMérieux Inc, https://www.biomerieux-usa.com). Pure cultures of *C. auris* isolates were stored at −80°C.

The Qiagen EZ1 Blood and Tissue Kit and the EZ1 Advanced XL instrument were used to extract genomic DNA from pure isolates of *C. auris*. Extracted DNA was quantified with the Qubit 1X dsDNA HS assay using the Qubit 3.0 Fluorometer (ThermoFisher Scientific, https://www.thermofisher.com). Acceptable quantities of DNA were ≥0.04 ng/µL.

DNA was diluted in water to obtain concentrations within the range of 100–500 ng in 30µL. Library preparation was performed using the Nextera DNA Flex Library Prep Kit (Illumina, https://www.illumina.com) according to manufacturer's instructions. Transposome

mediated fragmentation DNA was amplified using a limited-cycle PCR set according to the input DNA concentrations. A library cleanup step was performed using a 2-step bead purification procedure. Libraries were quantified, as described above, using Qubit; acceptable concentrations were ≥0.1 ng/μL. Following quantification, the Agilent DNA 1000 Kit and Agilent 2100 Bioanalyzer instrument (Agilent, https://www.agilent.com) were used to analyze the average band size of the libraries with an acceptable range of 300–900 bp.

Acceptable libraries were normalized to 2nM or 4nM concentrations for the Illumina MiSeq version 2 or version 3 reagent kit, respectively. Five microliters of each sample were pooled, denatured, and diluted; 600μL were loaded into the reagent cartridge. The Illumina MiSeq System was used to produce 250 bp paired-end reads. Data were uploaded to the Illumina BaseSpace cloud and de-multiplexed.

Six *C. auris* isolates from five patients were sequenced; sequences were uploaded to GenBank under the BioProject: PRJNA672695. UCLA_A1 was isolated from an inguinal/axillary swab of patient A on October 8, 2019 (GenBank accession no. SRR12916694). UCLA_A2 was isolated from a tracheal suction specimen of patient A on October 8, 2019 (GenBank accession no. SRR12916693). UCLA_C1 was isolated from an inguinal/axillary swab of patient C on August 12, 2020 (GenBank accession no. SRR12916692). UCLA_D1 was isolated from an inguinal/axillary swab of patient D on August 19, 2020 (GenBank accession no. SRR12916691). UCLA_E1 was isolated from an inguinal/axillary swab of patient E on August 31, 2020 (GenBank accession no. SRR12916690). UCLA_F1 was isolated from a right pleural fluid specimen of patient F on September 3, 2020 (GenBank accession no. SRR12916689).

Bioinformatics and Phylogenetic Analyses

CLC Genomics Workbench version 12.0.3 was used to pair, trim, and map the sequence reads. Reads were mapped to a reference *C. auris* complete genome (ATCC MYA-5022, GenBank accession no. CP043531); this strain is part of Clade III. The number of sequence reads ranged from 1,056,808–6,212,112. The percent of reads mapped to the reference genome ranged from 93.82%–96.33%. The percent of the reference genome with at least 10× coverage of mapped reads ranged from 91.90%–97.74%.

K-mer analyses were done using the Create K-mer Tree tool in CLC Genomics Workbench. K-mer's of 16 nt lengths were used; Jensen-Shannon Divergence was used to

construct the phylogenetic trees. The K-mer trees are displayed as circular phylograms. Single nucleotide polymorphism (SNP) analyses were done using the Basic Variant Detection tool in CLC Genomics Workbench. This tool was used to create a variant track following read mapping to the reference genome. The variant tracks for each isolate were then compared using the Create SNP Tree tool. SNP trees were created using maximum-likelihood algorithms.

Sequence reads were also mapped to the *erg11* and *fks1* genes of the reference genome. For *erg11*, the percent of the reference genome with ≥10× coverage of mapped reads ranged from 84.83%–100%. For *fks1*, the percent of the reference genome with ≥10× coverage of mapped reads ranged from 86.71%–100%. Using the Extract Consensus Sequence tool, the consensus sequences for both genes were determined. Consensus sequences were then exported from CLC Genomics Workbench and imported into Geneious Prime (Geneious, https://www.geneious.com) version 2020.0.3 for further analysis. The consensus sequence and the translated amino acid sequence of both genes for each isolate were compared to the reference sequence using the Geneious Alignment tool.

Additional Genomes Used

An additional 251 previously analyzed genomes of *C. auris* were used in the k-mer and SNP analyses (Appendix 2 Table, https://wwwnc.cdc.gov/EID/article/27/5/20-4361-App2.xlsx). Collection dates range from 2009–2020 from a variety of specimen sources and geographic locations (*1*–*8*).

References

1. Chow NA, Gade L, Tsay SV, Forsberg K, Greenko JA, Southwick KL, et al.; US *Candida auris* Investigation Team. Multiple introductions and subsequent transmission of multidrug-resistant *Candida auris* in the USA: a molecular epidemiological survey. Lancet Infect Dis. 2018;18:1377–84. PubMed https://doi.org/10.1016/S1473-3099(18)30597-8

2. Chow NA, Muñoz JF, Gade L, Berkow EL, Li X, Welsh RM, et al. Tracing the evolutionary history and global expansion of *Candida auris* using population genomic analyses. mBiol 2020;11:e03364–19. PubMed https://doi.org/10.1128/mBio.03364-19

3. Escandón P, Chow NA, Caceres DH, Gade L, Berkow EL, Armstrong P, et al. Molecular epidemiology of *Candida auris* in Colombia reveals a highly related, countrywide colonization with regional patterns in amphotericin B resistance. Clin Infect Dis. 2019;68:15–21. PubMed

4. Hamprecht A, Barber AE, Mellinghoff SC, Thelen P, Walther G, Yu Y, et al.; German Candida auris Study Group. *Candida auris* in Germany and previous exposure to foreign healthcare. Emerg Infect Dis. 2019;25:1763–5. PubMed https://doi.org/10.3201/eid2509.190262

5. Heath CH, Dyer JR, Pang S, Coombs GW, Gardam DJ. *Candida auris* sternal osteomyelitis in a man from Kenya visiting Australia, 2015. Emerg Infect Dis. 2019;25:192–4. PubMed https://doi.org/10.3201/eid2501.181321

6. Lockhart SR, Etienne KA, Vallabhaneni S, Farooqi J, Chowdhary A, Govender NP, et al. Simultaneous emergence of multidrug-resistant *Candida auris* on 3 continents confirmed by whole-genome sequencing and epidemiological analyses. Clin Infect Dis. 2017;64:134–40. PubMed https://doi.org/10.1093/cid/ciw691

7. Rhodes J, Abdolrasouli A, Farrer RA, Cuomo CA, Aanensen DM, Armstrong-James D, et al. Genomic epidemiology of the UK outbreak of the emerging human fungal pathogen *Candida auris* [Erratum in: Emerg Microbes Infect. 2018;7:104]. Emerg Microbes Infect. 2018;7:43. PubMed https://doi.org/10.1038/s41426-018-0045-x

8. National Center for Biotechnology Information. NCBI pathogen detection. 2021 [cited 2020 Nov 1]. https://www.ncbi.nlm.nih.gov/pathogens/isolates/#Candida%20auris

Appendix Table 1. Single nucleotide polymorphism analysis of *Candida auris* isolates, United States, 2013–2020*

Isolate†	UCLA_A1	UCLA_A2	UCLA_C1	UCLA_D1	UCLA_E1	UCLA_F1	Clade III	Clade III	Clade I	Clade II	Clade IV
UCLA_A1	–	3	12	10	7	77	47	66	24,374	35,804	95,891
UCLA_A2	3	–	11	9	6	78	46	65	24,373	35,803	95,890
UCLA_C1	12	11	–	4	7	77	51	74	24,382	35,808	95,893
UCLA_D1	10	9	4	–	5	79	53	72	24,380	35,810	95,894
UCLA_E1	7	6	7	5	–	76	50	69	24,377	35,807	95,894
UCLA_F1	77	78	77	79	76	–	117	139	24,404	35,797	95,918
Clade III	47	46	51	53	50	117	–	61	24,370	35,792	95,884
Clade III	66	65	74	72	69	139	61	–	24,385	35,814	95,906
Clade I	24,374	24,373	24,382	24,380	24,377	24,404	24,370	24,385	–	36,606	95,700
Clade II	35,804	35,803	35,808	35,810	35,807	35,797	35,792	35,814	36,606	–	97,644
Clade IV	95,891	95,890	95,893	95,894	95,894	95,918	95,884	95,906	95,700	97,644	–

*Reads were mapped to the *C. auris* reference genome (GenBank accession no. CP043531); consensus sequences were extracted from the alignments. GenBank accession no. SRR12073484 was isolated from Florida; GenBank accession no.SRR7909359 was isolated from Indiana.
†Clade III represented by GenBank accession nos. SRR12073484 and SRR7909359; clade I represented by GenBank accession no. SRR7909162; clade II represented by GenBank accession no. SRR7909356; clade IV represented by GenBank accession no. SRR7909221.

Appendix Table 2. Nucleotide and amino acid variants of *Candida auris* isolates, Los Angeles, California, USA, 2019–2020*

	erg11		*fks1*	
C. auris Isolate	Nucleotide pairwise identity, %	Protein sequence variants	Nucleotide pairwise identity, %	Protein sequence variants
UCLA_A1	99.6	p.V125A<br>p.F126L	99.9	p.I1572L
UCLA_A2	99.6	p.V125A<br>p.F126L	99.9	p.I1572L
UCLA_C1	99.6	p.V125A<br>p.F126L	99.9	p.I1572L
UCLA_D1	99.6	p.V125A<br>p.F126L	99.9	p.I1572L
UCLA_E1	99.6	p.V125A<br>p.F126L	99.9	p.I1572L
UCLA_F1	99.6	p.V125A<br>p.F126L	99.8	p.I1095L<br>p.I1572L

*Nucleotide pairwise identity to *C. auris* strain ATCC MYA-5002 (GenBank accession no. CP043531). Protein translation and variant analysis conducted using Geneious Prime (Geneious, https://www.geneious.com).

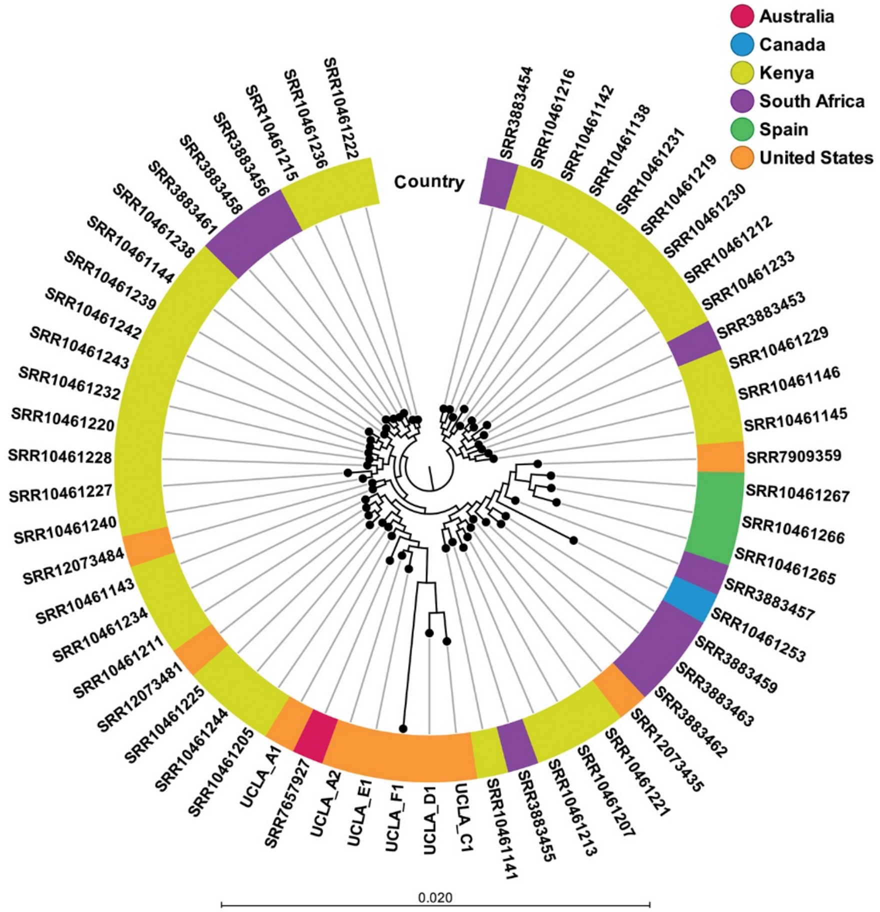

Appendix Figure. Global phylogenetic analysis of *Candida auris* clade III isolates. Analysis based on k-mers of 261 publicly available *C. auris* sequences (Appendix 2 Table 1, https://wwwnc.cdc.gov/EID/article/27/5/20-4361-App2.xlsx). Colors indicate country of origin. Scale indicates branch lengths within the tree.
